# Supplementary figures and images for: CEP78 functions downstream of CEP350 to control biogenesis of primary cilia by negatively regulating CP110 levels
Source: eLife. 2021 Jul 14;10:e63731. doi: 10.7554/eLife.63731 (PMC8354638; doi:10.7554/eLife.63731)

Figure 1-source data 1

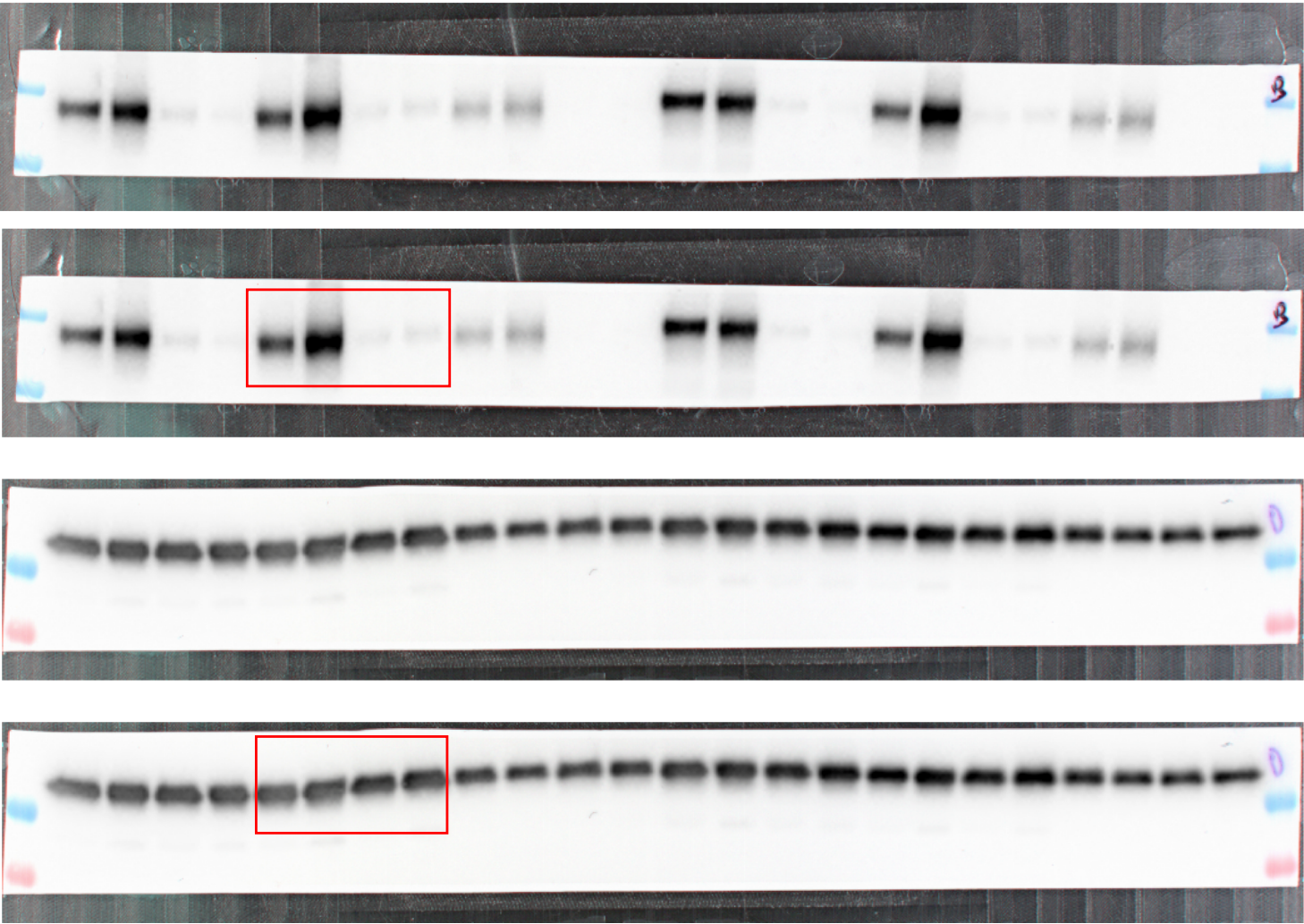

Supplement: Figure 1—source data 1. — The upper blots are labeled with pRb antibody and lower blots with GAPDH antibody. [file elife-63731-fig1-data1.pdf]

Figure 1-source data 2

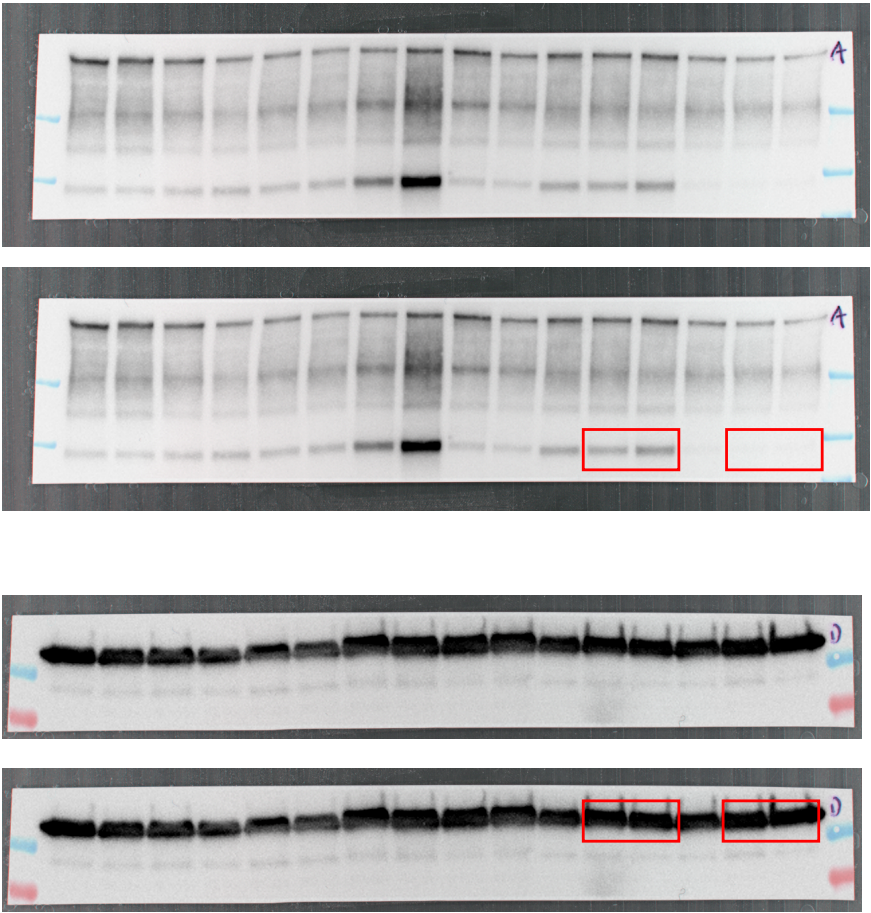

Supplement: Figure 1—source data 2. — The upper blots are labeled with pRb antibody and lower blots with GAPDH antibody. [file elife-63731-fig1-data2.pdf]

Figure 1-figure supplement 1-source data 1

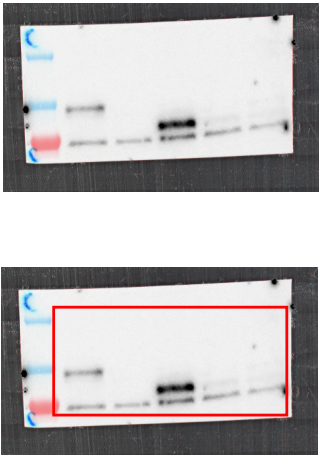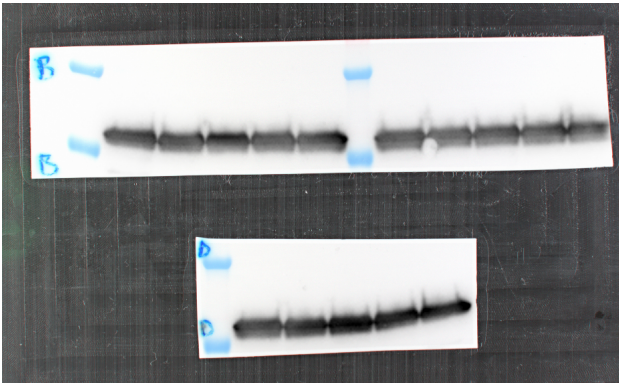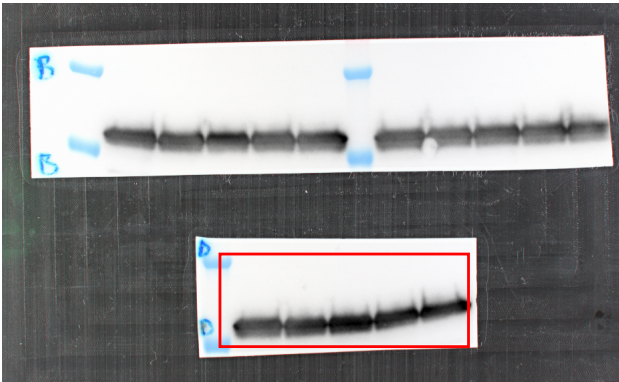

Supplement: Figure 1—figure supplement 1—source data 1. — Left, CEP78 blot; right, GAPDH blot. [file elife-63731-fig1-figsupp1-data1.pdf]

Figure 2-figure supplement 1-source data 1

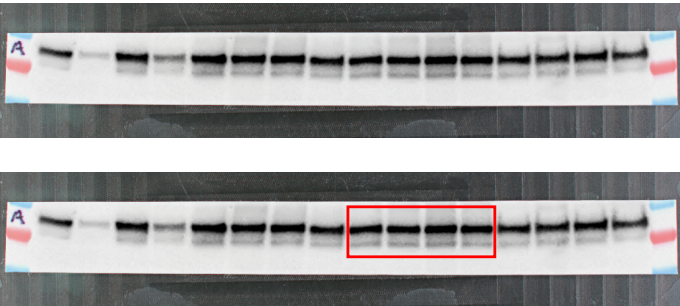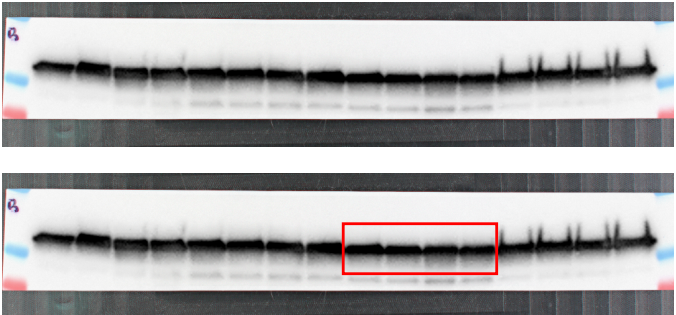

Supplement: Figure 2—figure supplement 1—source data 1. — Left, FLAG blot; right, GAPDH blot. [file elife-63731-fig2-figsupp1-data1.pdf]

Figure 2-figure supplement 2-source data 1

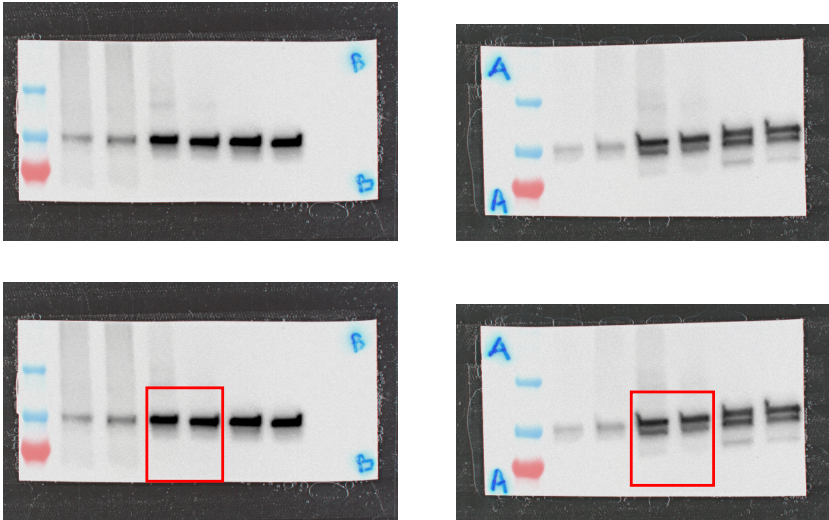

Supplement: Figure 2—figure supplement 2—source data 1. — Left, FLAG blot; right, CEP78 blot. [file elife-63731-fig2-figsupp2-data1.pdf]

Figure 2-figure supplement 3-source data 1

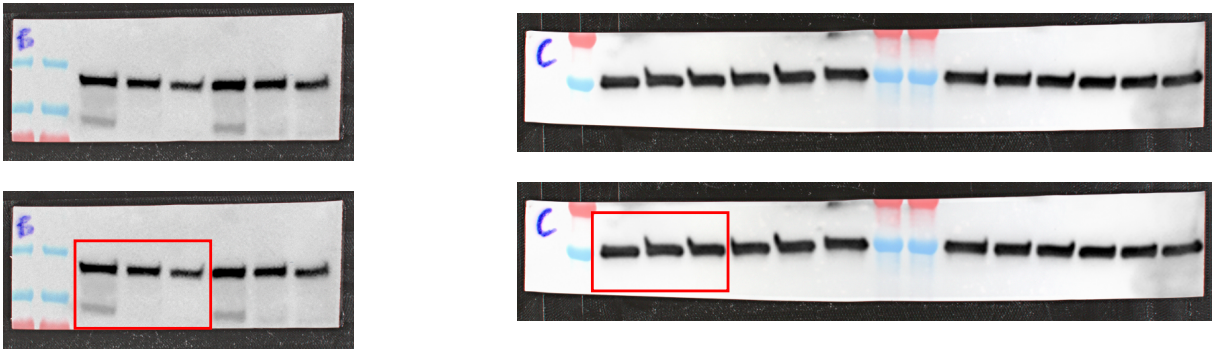

Supplement: Figure 2—figure supplement 3—source data 1. — Left, CEP78 blot; right, α-tubulin blot. [file elife-63731-fig2-figsupp3-data1.pdf]

Figure 3-source data 2 (panel B)

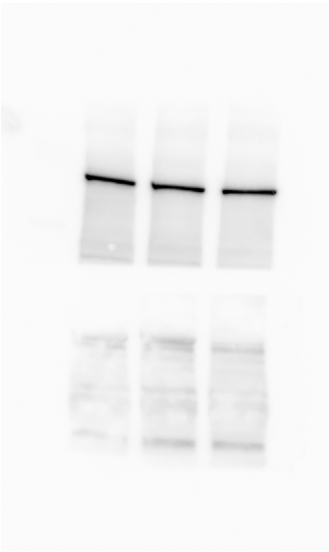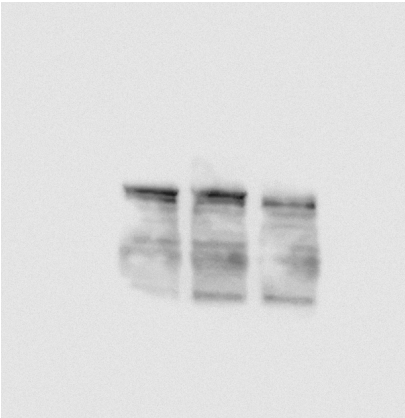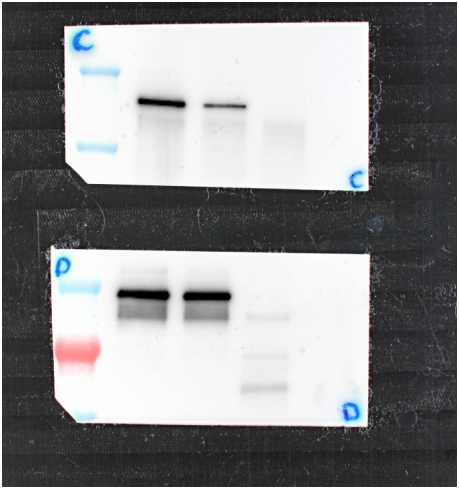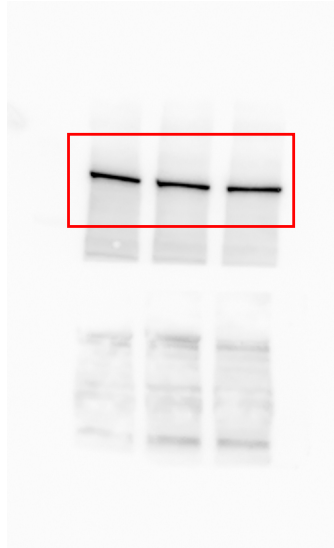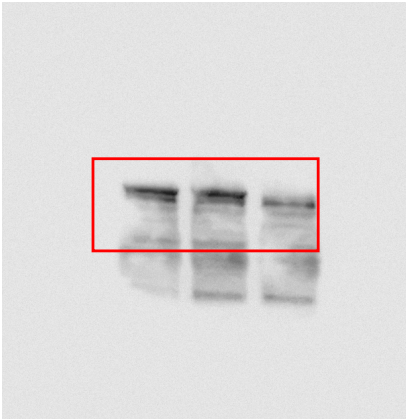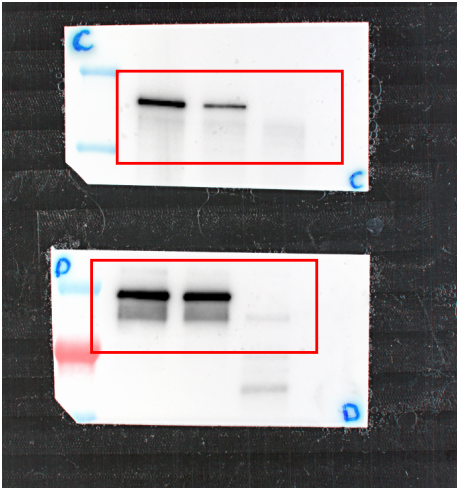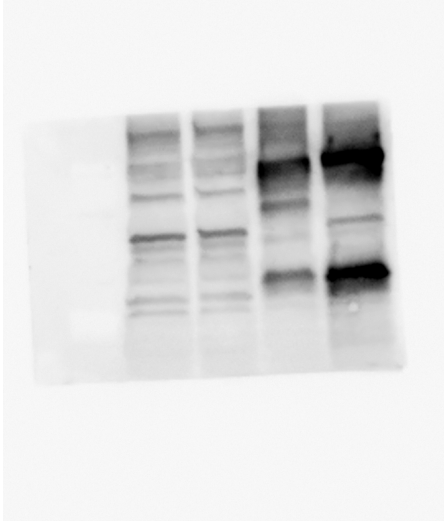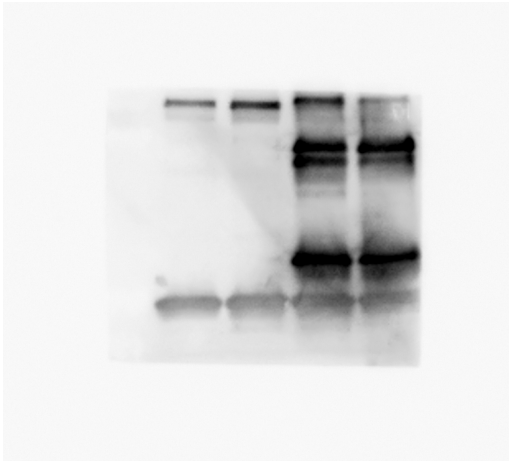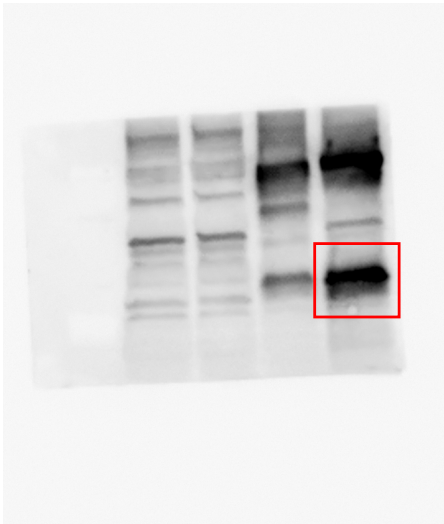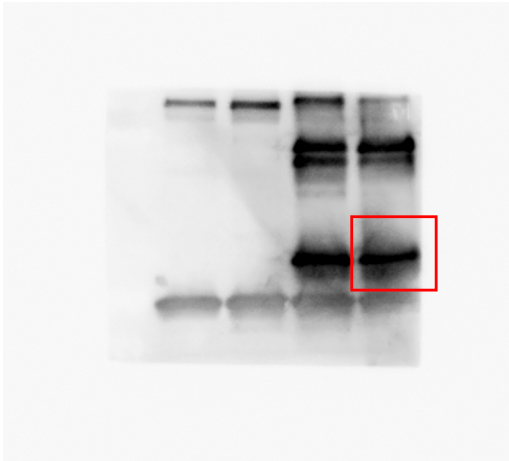

Supplement: Figure 3—source data 2. — Upper left, input Myc blot; upper middle, input 100 kDa FLAG blot; upper right, immunoprecipitation (IP) Myc and 100 kDa FLAG blots; bottom left, input 35 kDa FLAG blot; bottom right, IP 35 kDa FLAG blot. [file elife-63731-fig3-data2.pdf]

Figure 3-source data 3 (panel C)

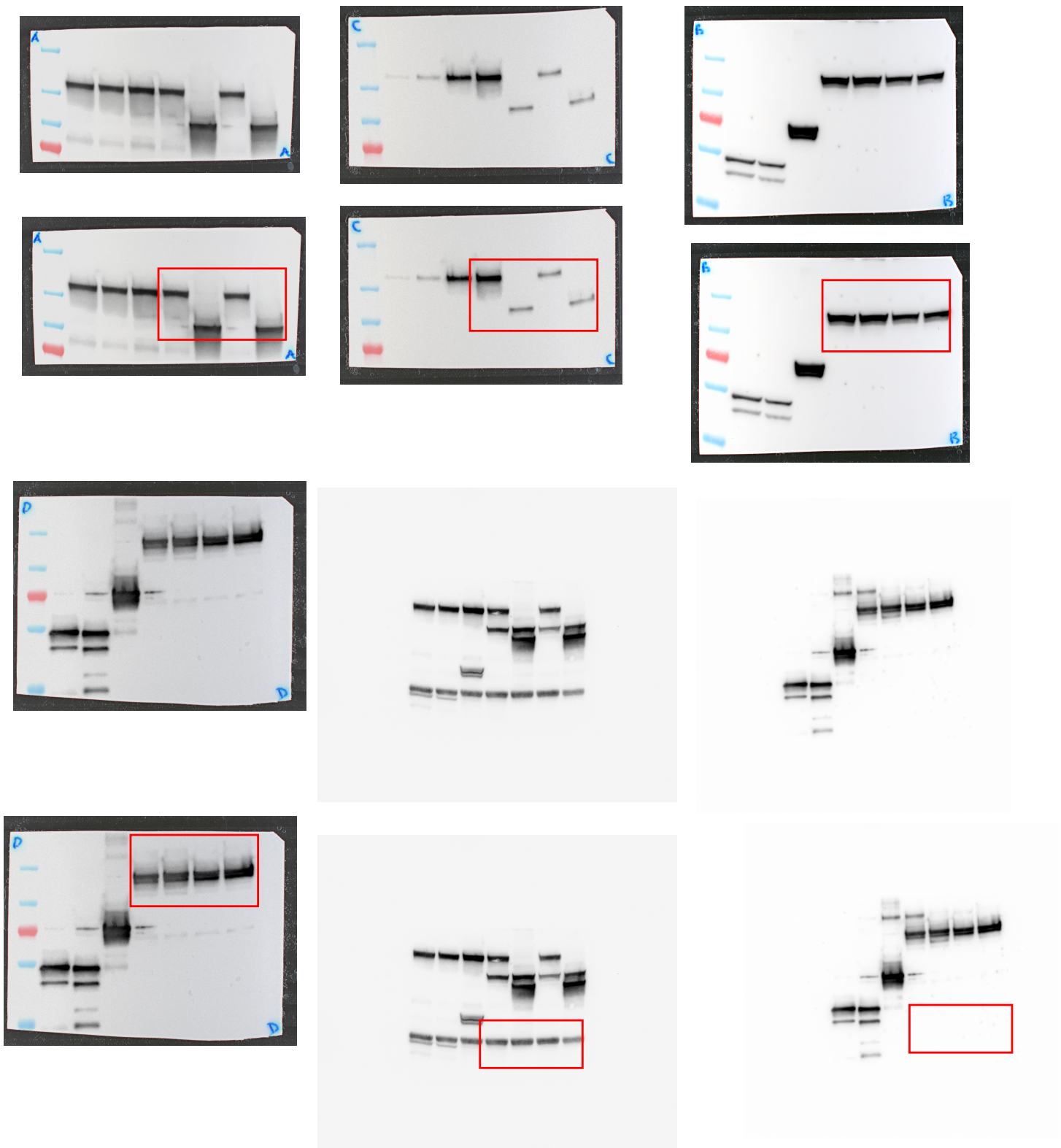

Supplement: Figure 3—source data 3. — Upper left, input Myc blot; upper middle, immunoprecipitation (IP) Myc blot; upper right, input GFP blot; bottom left, IP GFP blot; bottom middle, input FOP blot; bottom right, IP FOP blot. [file elife-63731-fig3-data3.pdf]

Figure 3-source data 4 (panel D)

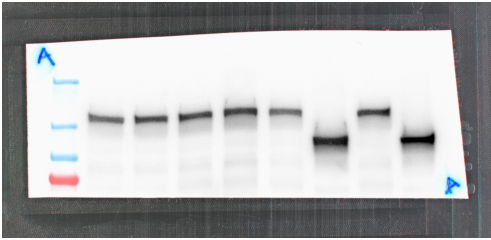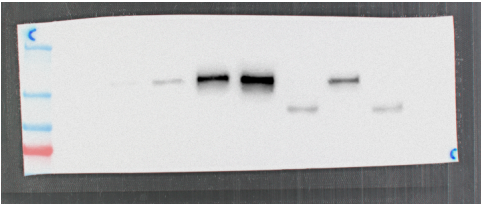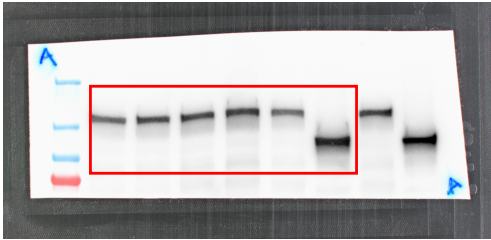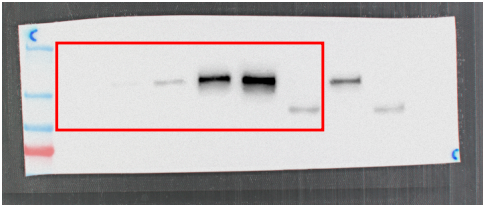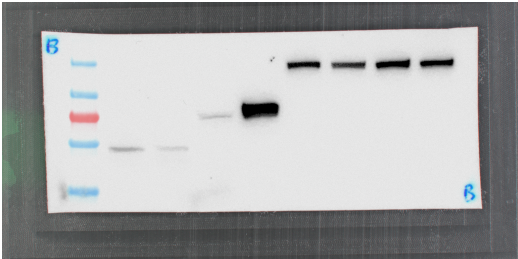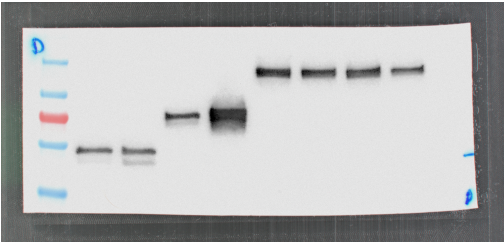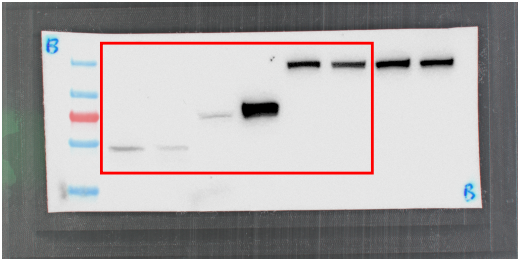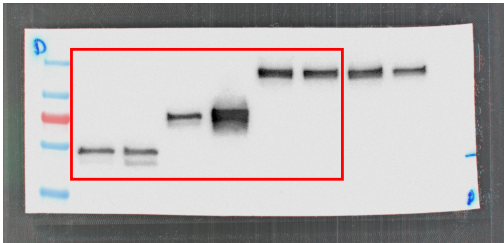

Supplement: Figure 3—source data 4. — Upper left, input Myc blot; upper right, immunoprecipitation (IP) Myc blot; lower left, input GFP blot; lower right, IP GFP blot. [file elife-63731-fig3-data4.pdf]

Figure 3-figure supplement 1-source data 1

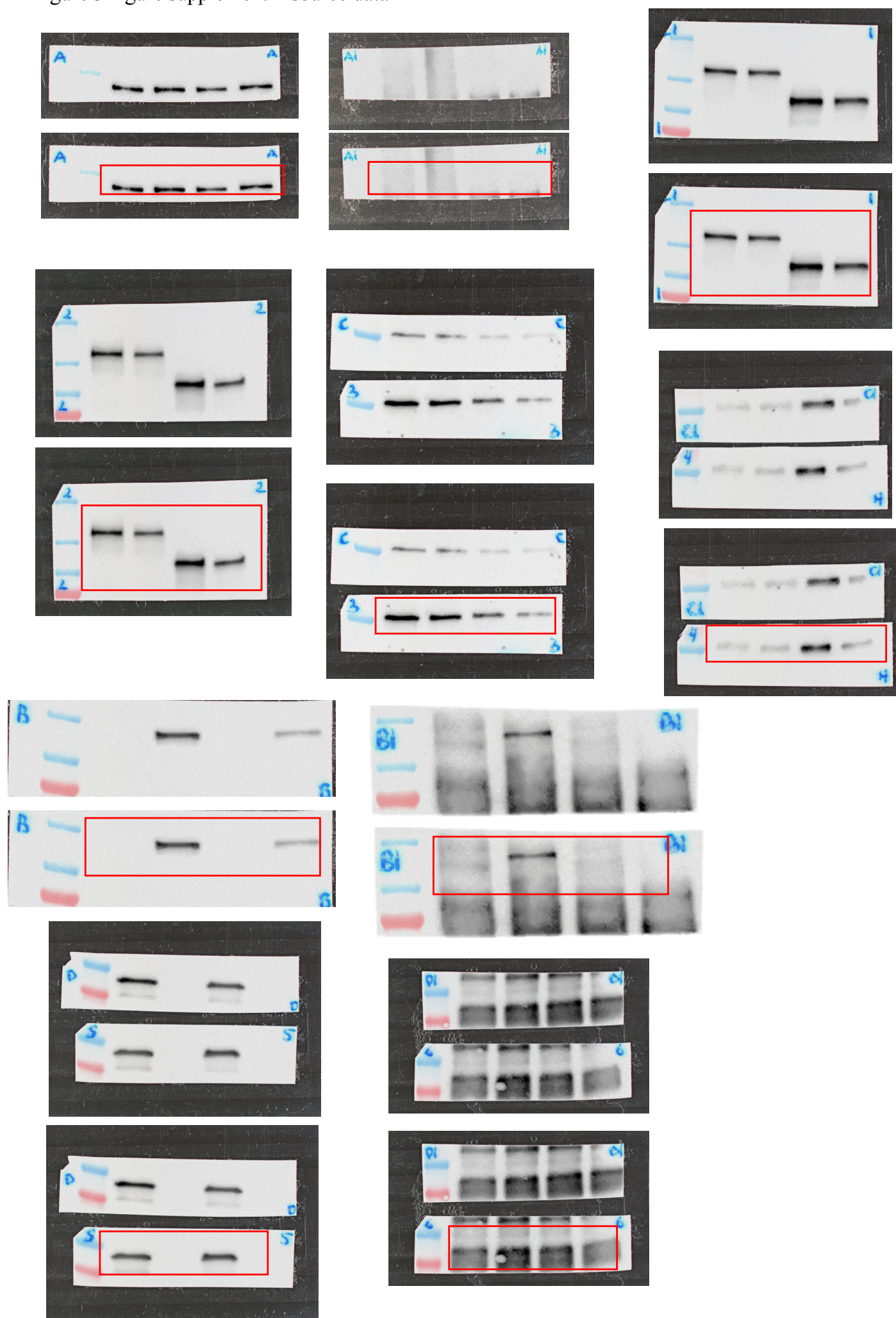

Supplement: Figure 3—figure supplement 1—source data 1. — Top row from left to right: input VPRBP blot, immunoprecipitation (IP) VPRBP blot, input Myc blot. Second row from left to right: IP Myc blot, input FOP blot, IP FOP blot. Third row from left to right: input GFP blot (upper), IP GFP blot (upper). Fourth row left to right: input GFP blot (lower), IP GFP blot (lower). [file elife-63731-fig3-figsupp1-data1.pdf]

Figure 3-figure supplement 1-source data 2

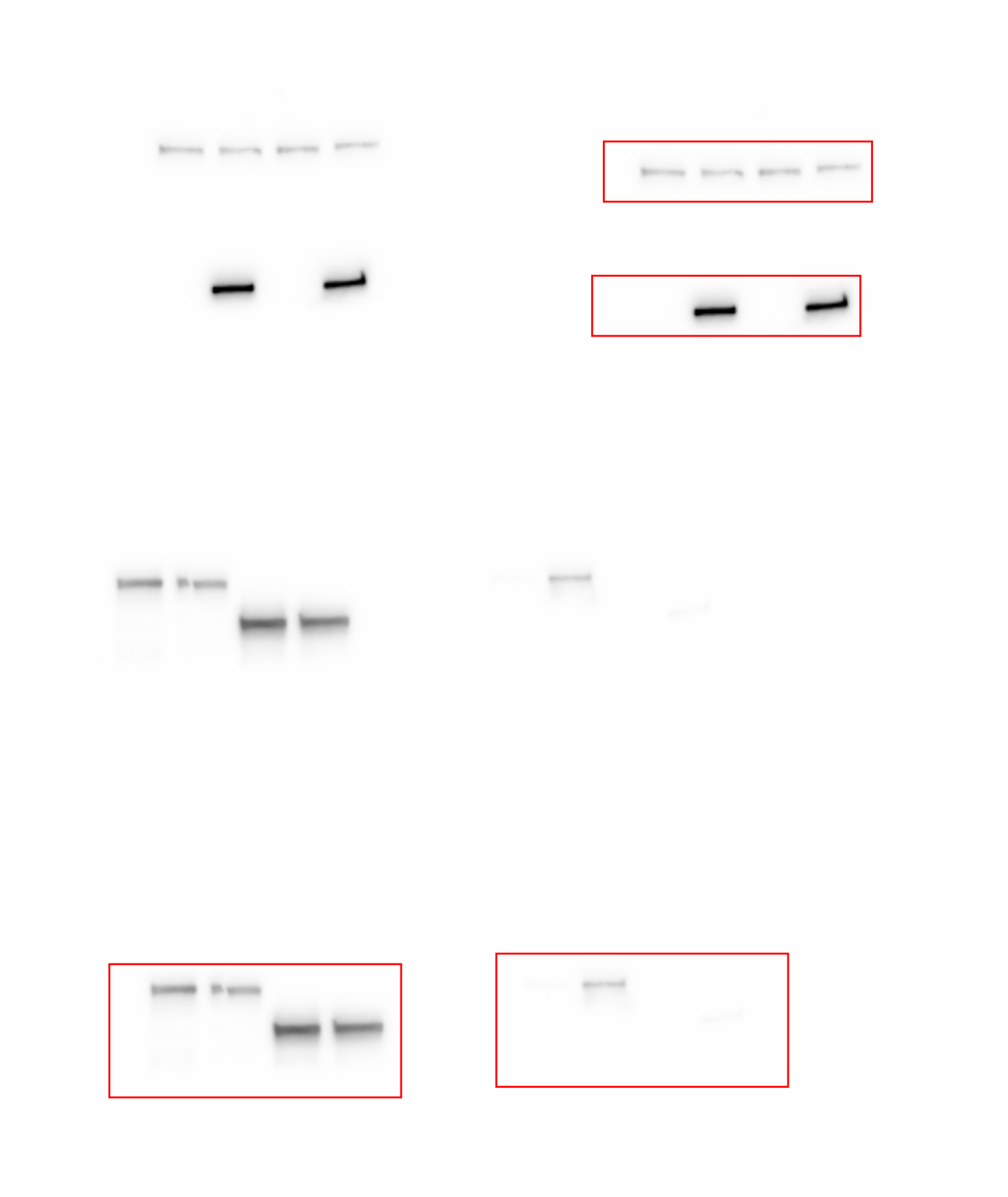

Figure 3-figure supplement 1-source data 2

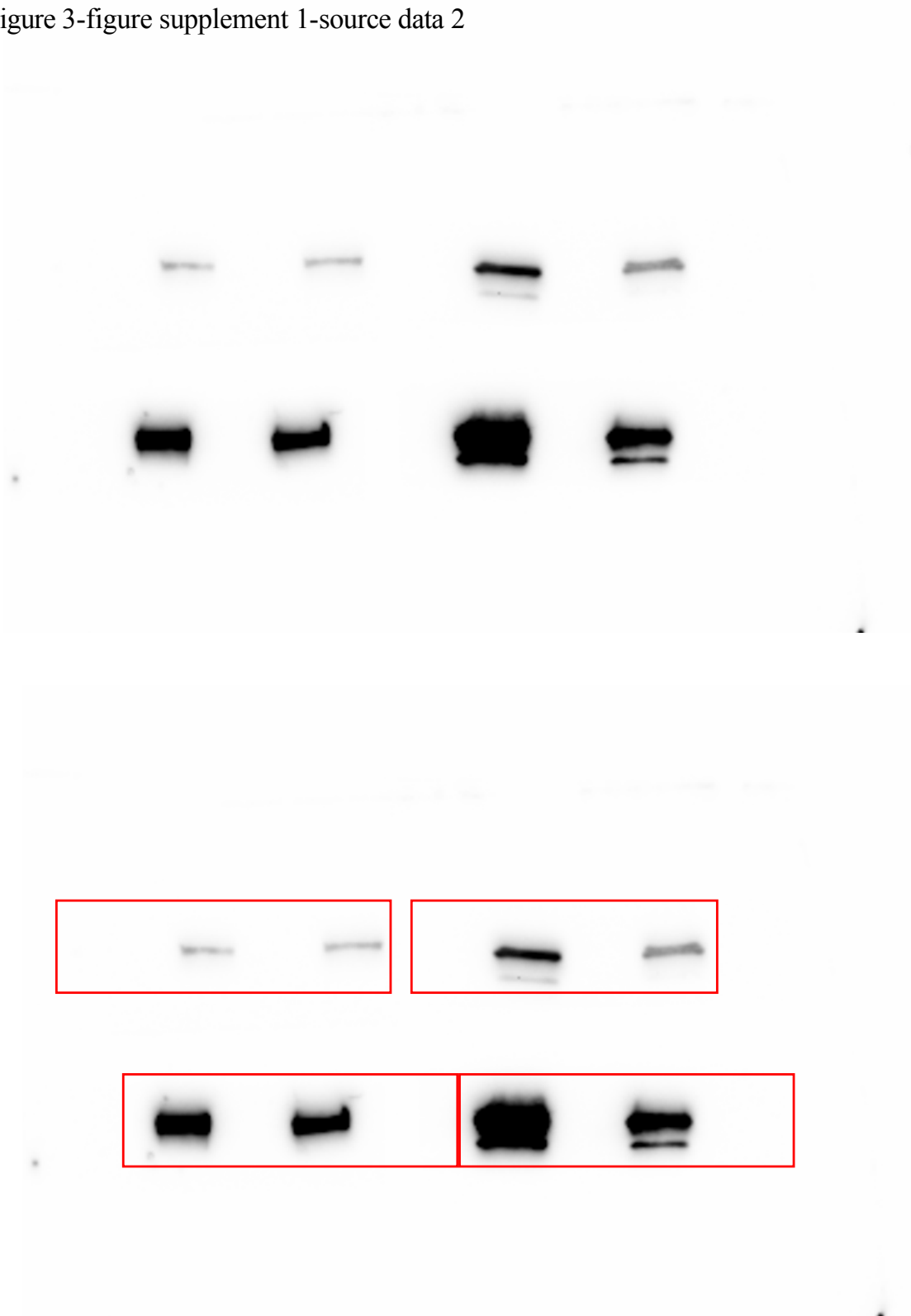

Supplement: Figure 3—figure supplement 1—source data 2. — Top row, input VPRBP blot; second row, immunoprecipitation (IP) VPRBP blot; third row left, input Myc blot; third row right, IP Myc blot; fourth row left, input GFP blots; fourth row right, IP GFP blots. [file elife-63731-fig3-figsupp1-data2.pdf]

Figure 4-source data 1

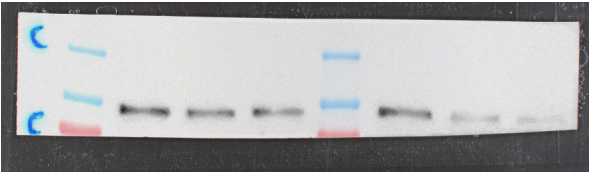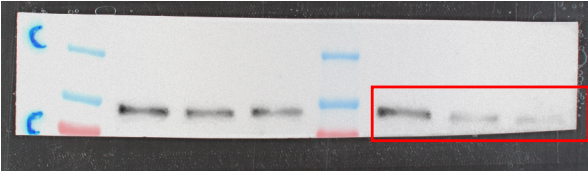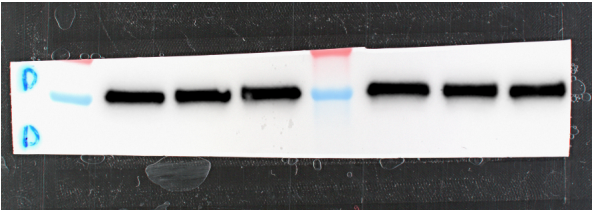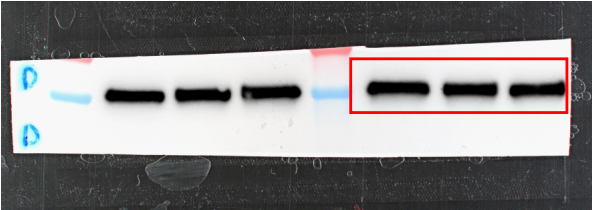

Supplement: Figure 4—source data 1. — Left, CEP78 blot; right, α-tubulin blot. [file elife-63731-fig4-data1.pdf]

Figure 6-source data 1

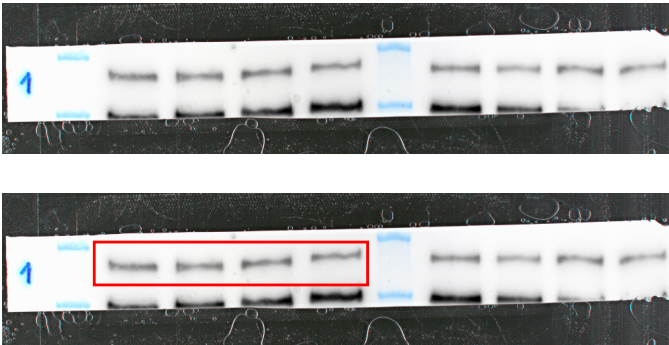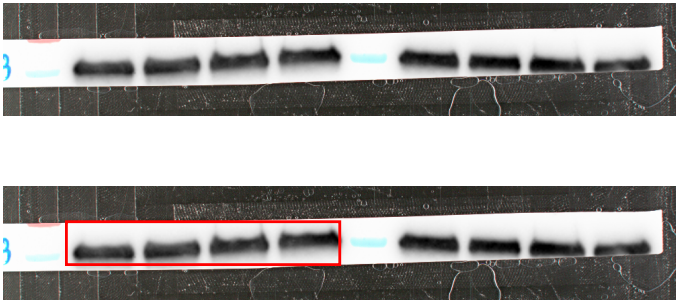

Supplement: Figure 6—source data 1. — Left, VPRBP blot; right, α-tubulin blot. [file elife-63731-fig6-data1.pdf]

Figure 7-source data 1

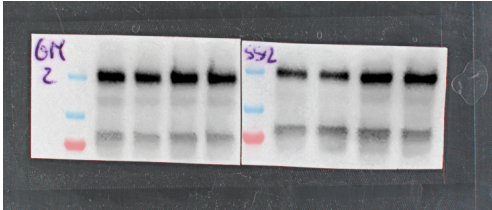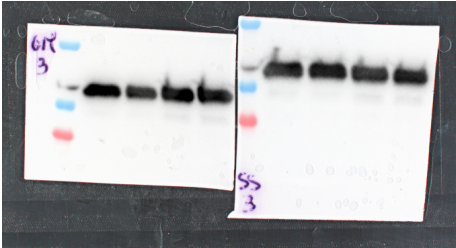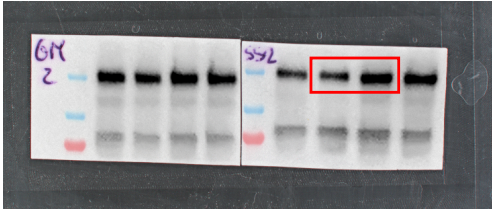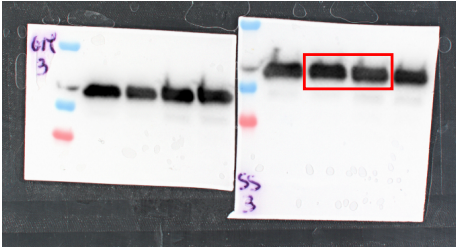

Supplement: Figure 7—source data 1. — Left, CP110 blot; right, GAPDH blot. [file elife-63731-fig7-data1.pdf]

Figure 7-figure supplement 1-source data 1

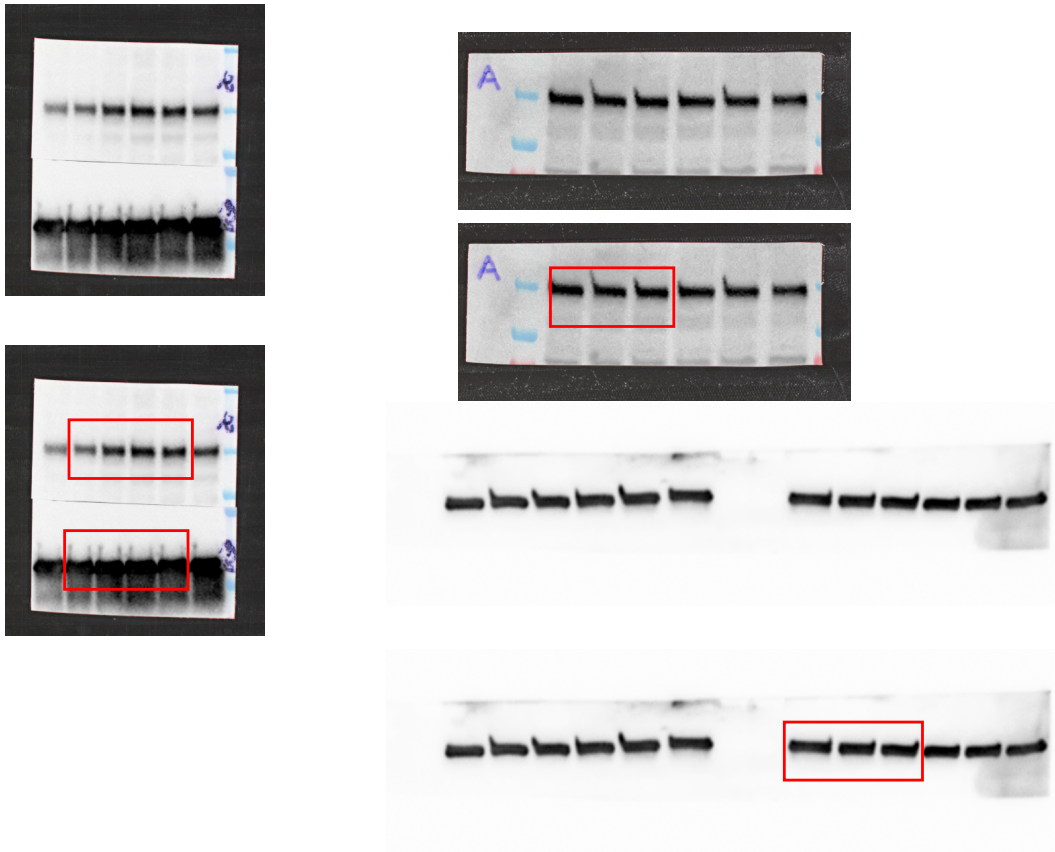

Supplement: Figure 7—figure supplement 1—source data 1. — Left, CP110 (upper) and GAPDH blots in (A). Right, CP110 (upper) and α−tubulin (lower) blots in (C). [file elife-63731-fig7-figsupp1-data1.pdf]

Figure 7-figure supplement 2-source data 1

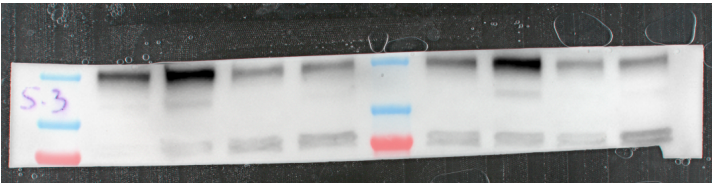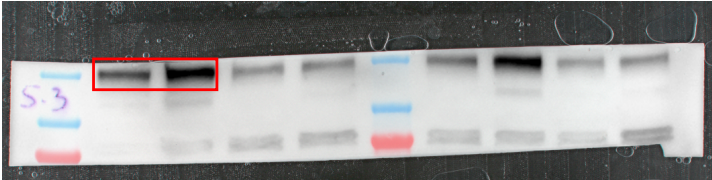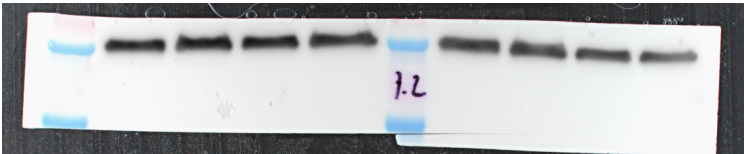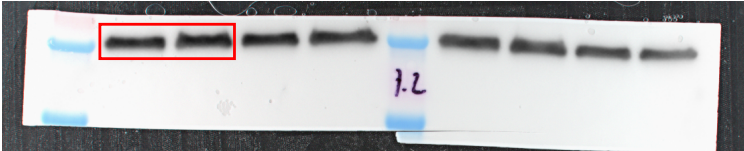

Supplement: Figure 7—figure supplement 2—source data 1. — Top, CP110 blot; bottom, α-tubulin blot. [file elife-63731-fig7-figsupp2-data1.pdf]

Figure 7-figure supplement 4-source data 1

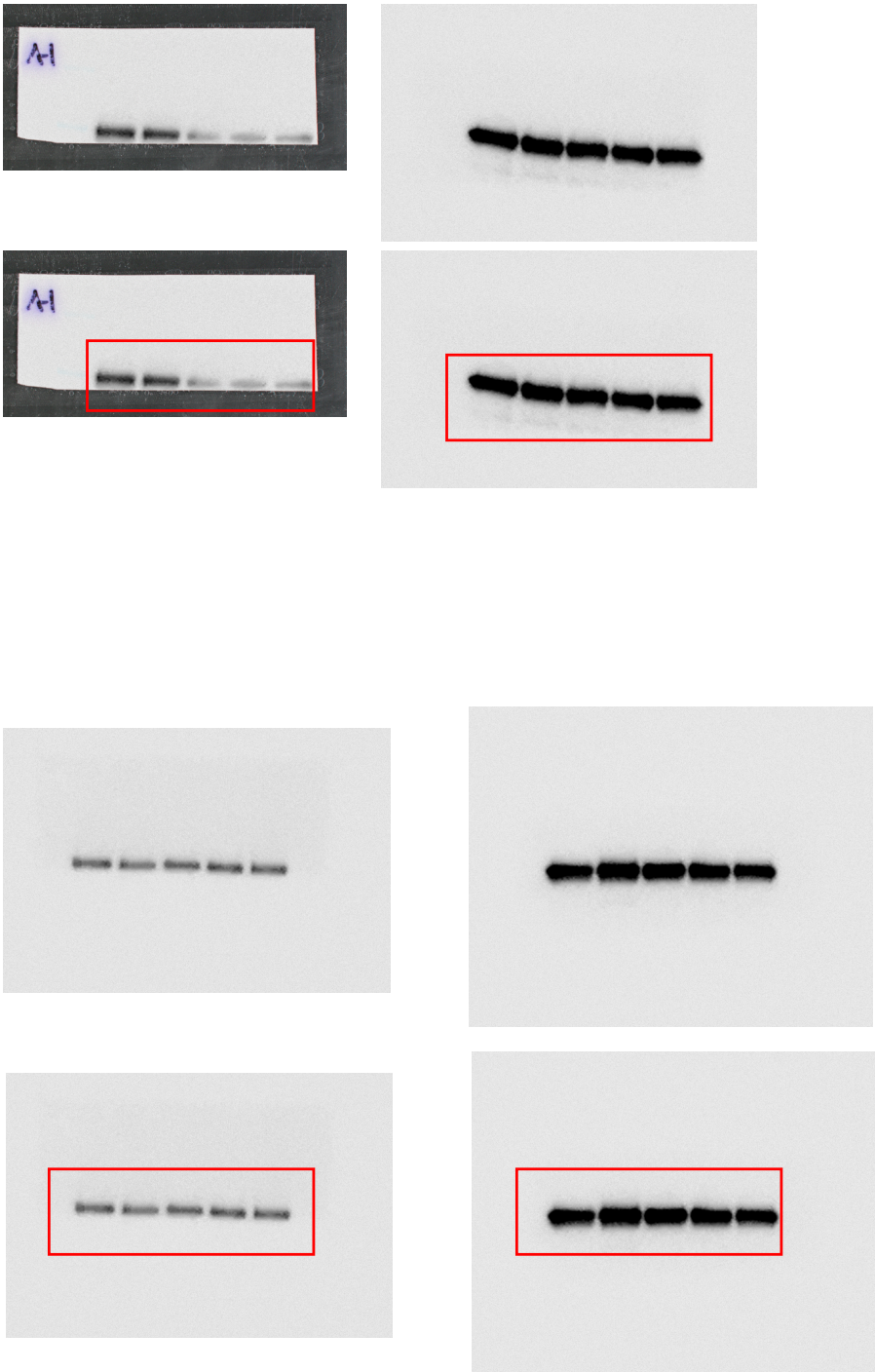

Supplement: Figure 7—figure supplement 4—source data 1. — Upper left, wildtype (WT) CP110 blot; upper right, WT GAPDH blot. Lower left, CEP78 knockout (KO) CP110 blot; lower right, CEP78 KO GAPDH blot. [file elife-63731-fig7-figsupp4-data1.pdf]

Figure 8-source data 1

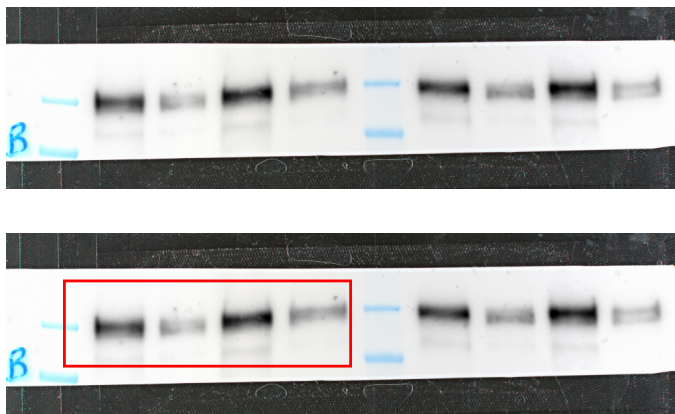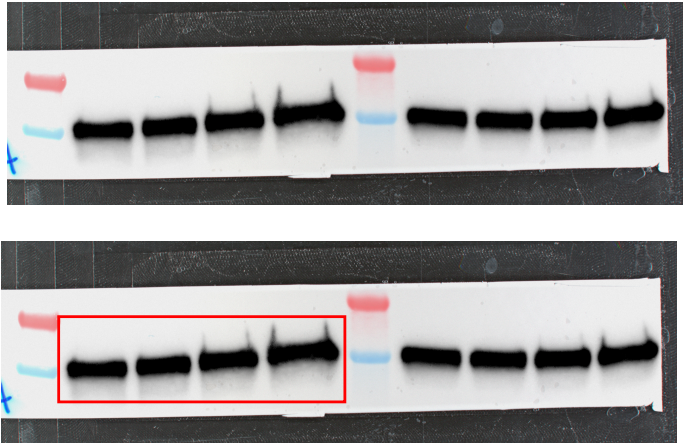

Supplement: Figure 8—source data 1. — Left, CP110 blot; right, α-tubulin blot. [file elife-63731-fig8-data1.pdf]
